# Development of Coral Investigation System Based on Semantic Segmentation of Single-Channel Images

**DOI:** 10.3390/s21051848

**Published:** 2021-03-06

**Authors:** Hong Song, Syed Raza Mehdi, Yangfan Zhang, Yichun Shentu, Qixin Wan, Wenxin Wang, Kazim Raza, Hui Huang

**Affiliations:** Department of Ocean Engineering and Technology, Ocean College, Zhejiang University, Zheda Road 1, Zhoushan 316021, China; hongsong@zju.edu.cn (H.S.); 12034098@zju.edu.cn (S.R.M.); 21934131@zju.edu.cn (Y.Z.); styc@zju.edu.cn (Y.S.); 21834179@zju.edu.cn (Q.W.); 21834180@zju.edu.cn (W.W.); kazim14@zju.edu.cn (K.R.)

**Keywords:** coral, semantic segmentation, spectral imaging, convolutional neural networks, deep learning, image processing

## Abstract

Among aquatic biota, corals provide shelter with sufficient nutrition to a wide variety of underwater life. However, a severe decline in the coral resources can be noted in the last decades due to global environmental changes causing marine pollution. Hence, it is of paramount importance to develop and deploy swift coral monitoring system to alleviate the destruction of corals. Performing semantic segmentation on underwater images is one of the most efficient methods for automatic investigation of corals. Firstly, to design a coral investigation system, RGB and spectral images of various types of corals in natural and artificial aquatic sites are collected. Based on single-channel images, a convolutional neural network (CNN) model, named DeeperLabC, is employed for the semantic segmentation of corals, which is a concise and modified deeperlab model with encoder-decoder architecture. Using ResNet34 as a skeleton network, the proposed model extracts coral features in the images and performs semantic segmentation. DeeperLabC achieved state-of-the-art coral segmentation with an overall mean intersection over union (IoU) value of 93.90%, and maximum F1-score of 97.10% which surpassed other existing benchmark neural networks for semantic segmentation. The class activation map (CAM) module also proved the excellent performance of the DeeperLabC model in binary classification among coral and non-coral bodies.

## 1. Introduction

Corals are a significant part of the marine ecosystem, with high primary productivity, providing habitation with ample nourishment for various underwater organisms [1,2]. Despite its great importance in the marine ecosystem, corals and other organisms inhabiting the surroundings face unprecedented challenges. Because of the gradual degradation of coral resources, it is essential to put forward scientific programs to maintain corals, which embarks with the coral investigation phase. Coral monitoring based on manual optical detection through images and videos by divers is common in small-scale monitoring [3,4,5]. However, with varying depth, diving time, and the divers’ filming speed, it is difficult to carry out long-term and large-scale monitoring.

Satellite remote sensing, using spectral imaging, is commonly practiced for large-scale coral study [6,7]. Although the spectral feature of each spatial pixel can be extracted from the image. However, the remote sensing spatial resolution is finite, and a particular spatial pixel in the image may have a large imaging area with varying types of corals. In a heavily dense coral area, it is difficult to differentiate the spectral characteristics of different corals. Also, the limited propagation of natural light in seawater makes it challenging to detect the coral situation through remote sensing technology [8].

The coral distribution should be analyzed after the image data is collected irrespective of the scale of observation. Coral coverage zones in an image, as one of the essential statistical indicators, presents the proportion of coral in the investigated area, which can be obtained by calculating the number of spatial pixels occupied by corals and all the spatial pixels in the image. The segmentation result is that all the pixels belonging to corals are marked uniformly, and the remaining pixels are classified as background.

Traditional coral image data analysis mainly depends on the experts who master the knowledge of marine ecology. The pixel category is determined by comparing the morphological feature information of corals in the image [9]. This method has a high labor cost, and it is subjective to identification decisions by professionals.

In recent years, the rapid development of automatic judgment methods based on efficient computing power can be noticed. Data analysis for computer vision techniques, Refs. [10,11] such as recognition, classification, and semantic segmentation, can be carried rapidly without interruption with such automatic detection and judgment [12,13]. To date, several researches have been reported, using the application of such detection techniques combined with different imaging methods in the aquatic environment [14,15]. For the coral study, both spectral imaging and RGB imaging techniques have been used to get morphological features. Later, various machine learning algorithms have been utilized for automatic detection and segmentation as discussed in the following sections.

### 1.1. Segmentation Based on Spectral Features

The study of spectral features has inspired researchers to explore coral’s semantic segmentation in underwater spectral images. For this purpose, a line scanning Underwater Hyperspectral Imager (UHI) has been developed by Ectone, founded by the Norwegian University of Science and Technology. In 2016, the team collected spectral images of corals using UHI, later carried out semantic segmentation to analyze corals in the area. For semantic segmentation, different distance measurement techniques like Euclidean distance; Spectral Angle Measurement (SAM); Spectral Information Divergence (SID); and different classification algorithms such as K-Nearest Neighbor (KNN) and binary encoding classifier based on spectral feature differentiation, were tested. SAM distance measurement combined with k = 1 KNN classifier showed better results among other segmentation techniques [16].

In 2017, the Italian research team mounted an UHI on the remotely operated vehicle to conduct underwater spectral imaging of the South Asian sea. To classify among 11 types of coral reefs, a classification algorithm based on SAM was used pixel by pixel in the spectral image, which achieved an average accuracy of 72% for all categories [17].

In 2019, the first underwater spectral imaging of shallow coral habitat was carried out in Norway’s sea by an UHI carried by an unmanned ship. The UHI collected underwater spectral images of 400~700 nm at intervals of 3.5 nm. The collected spectral image data were manually labelled with pixel-level categories for training Support Vector Machine (SVM), which resulted in a maximum accuracy of 89% for semantic segmentation of corals [18].

In another research in 2019, deep-sea cold-water coral was exposed to different concentrations of 2-methylnaphthalene, resulting in different degrees of variation in its health. Spectral images of coral were collected before and after exposure to chemical and SVM was trained to distinguish among the coral in different health conditions. The semantic segmentation results showed that all the coral in the image with various health status were correctly classified [19].

### 1.2. Segmentation Based on RGB Image Features

In the last century, because of the limited availability of high computing power machines, digital image processing methods using mathematical calculations and topologies have been widely used for semantic segmentation based on image features. Among them, the simplest and fastest semantic segmentation is a simple threshold segmentation. Otsu’s method [20] of threshold selection has been used for semantic segmentation of corals with unvaried textures exposed to relatively uniform illumination throughout the image. For complex shapes, multi-threshold [21], adaptive threshold [22], and region growing algorithm are adopted to improve semantic segmentation effects. In 2012, Xu et al. improved the region-growing algorithm by adding tensor-based anisotropic analysis, which enhanced corals’ semantic segmentation effect [23].

Based on the gray level co-occurrence matrix, Shihavuddin et al. extracted the gray relationship of adjacent pixels, calculated the regional texture features, and further combined with SVM for semantic segmentation of seabed image containing coral. The segmentation results showed an overall accuracy of 83.7% [24].

Edge detection operators, such as Sobel operator [25], Robert operator [26], Prewitt operator [27], and Canny operator [28], are commonly used in semantic segmentation. Awalludin et al. improved Canny operator for coral edge detection by suppressing the interference of texture and imaging noise, to carry out edge detection and topological contour analysis for semantic segmentation of corals [29].

However, most of the above-mentioned techniques utilize low-level features in the images to perform semantic segmentation. Since AlexNet [30], CNN has made a significant breakthrough with remarkable improvement in semantic segmentation. Benefiting from skeleton networks, such as VGG [31], ResNet [32], and DenseNet [33], has improved the performance accuracy. The Fully Convolutional Network (FCN) proposed in 2015 is a classic algorithm for semantic segmentation [34]. Inspired by the excellent performance of FCN, Ronneberger et al. proposed UNet: a semantic segmentation network that improved the accuracy of FCN [35]. Multi-scale context aggregation by dilated convolutions proposed by Yu [36] and DeepLab series [37,38,39,40] by Google also provides new ideas for semantic segmentation. King et al. compared the semantic segmentation effect of the above networks on underwater RGB coral images. FCN achieved the lowest accuracy of 50.45% among the four algorithms. Benefiting from dilation convolution, the accuracy of improved Multi-scale context aggregation by dilated convolutions network is 62.84% and 64.90%, respectively. DeepLab V2 integrated with different scale dilation convolution achieved the highest accuracy of 67.70% [41].

To further expand the applications of deep CNNs with enhanced performance accuracy for underwater monitoring, this paper presents the analysis of the morphological information by a novel DeeperLabC model that can automatically extract spatial features and perform semantic segmentation of the coral image. The main contributions of this work are highlighted as follows.

1. First, a new CoralS dataset containing single-channel underwater coral images collected in natural underwater environment was constructed using RGB and spectral imaging techniques, developed in laboratory.
2. A deep CNN was modelled by fine-tuning deeplabv3+ model and adjusting ResNet34 backbone architecture for semantic segmentation of single-channel coral images. Depth to space module is added to the network to cope with the processing speed and memory usage of graphic processing unit (GPU).
3. CAM module was installed at the tail of CNN to enhance visualization of the model’s segmentation effects.
4. The fourth contribution is a comparison of the developed model, using the CoralS dataset, to benchmark CNN models; results showed that the proposed model achieved high accuracy. The comparison can be visualized through a GUI developed to perform coral image segmentation.

The rest of the paper is organized as follows: Section 2 introduces data and methods that include the construction of the coral dataset and the detailed structure of the proposed neural network model for coral semantic segmentation. Experimentations, including data preprocessing and network training, are outlined in Section 3. Segmentation effects, comparative analysis of the proposed model and GUI development are noted in Section 4. Lastly, synopsis of the work is presented in Section 5, which also draws the prospects of the presented study.

## 2. Methodology

Image semantic segmentation requires image dataset and a deep learning model which can properly converge the data for better segmentation results. For coral semantic segmentation, CoralS dataset is acquired by underwater RGB and spectral imaging techniques, later the images are preprocessed and manually annotated for further processing through a designed deep learning model for semantic segmentation of coral images. Image acquisition, processing, and the deep learning model are explained below.

### 2.1. CoralS Dataset Collection

Table 1 introduces CoralS dataset used for semantic segmentation of corals, which contains spectral images by Liquid Crystal Tunable Filter (LCTF) spectral imager [42], and RGB images taken with Single-lens Reflex (SLR) RGB camera (EOS, Canon, Tokyo, Japan). In addition to using a wide spectrum xenon lamp (HPLS-30-04, Thorlabs, Newton, NJ, USA), other sources such as sunlight, white lighting LED, and blue LED (FL-1, Nightsea, Lexington, MA, USA) were used as excitation light source.

**Figure A7:**
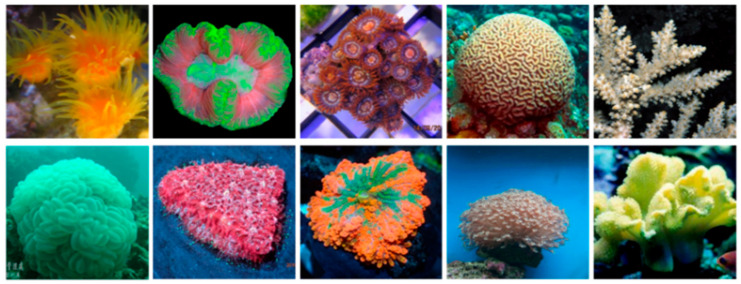
Examples of coral images that were collected through python crawler.

**Figure A8:**
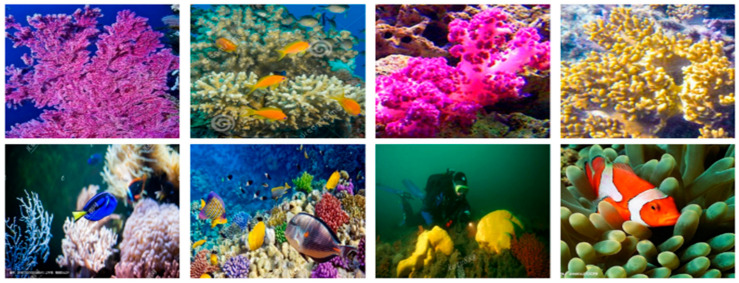
Coral images with complex background obtained by the web crawler.

Because of the limited number of coral species captured, python web crawler was used to get RGB images, shown in Figure A7, on the network as supplementary data samples to enrich coral morphological information. Crawler also collected coral images in more complex scenes as in Figure A8, containing various corals and other marine organisms. Python web crawler was executed in the following two steps:

1. ImageCrawler: obtain HTTP path of desired images;
2. ImageDownloader: download images to a local directory.

Parameters used for ImageCrawler and ImageDownloader were:

- engine: Baidu;
- keyword: Corals, Dead coral skeleton, and Acropora;
- n_scroll: It defines the number of scrolling in the browser;
- link_save_dir: It holds the directory to save web links of the images;
- image_save_dir: It defines a directory to save images on the local disk.

Crawler collected 500 images, including 400 positive sample images and 100 negative sample images, which makes up to 12% of the total image dataset. The overall quantitative analysis of the CoralS dataset is presented in Table 2.

**Figure A9:**
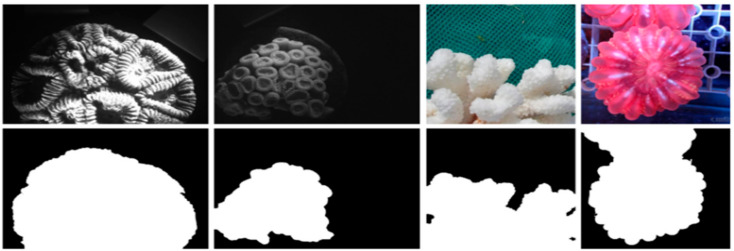
Examples of manual annotation of spectral and RGB images.

**Figure A10:**
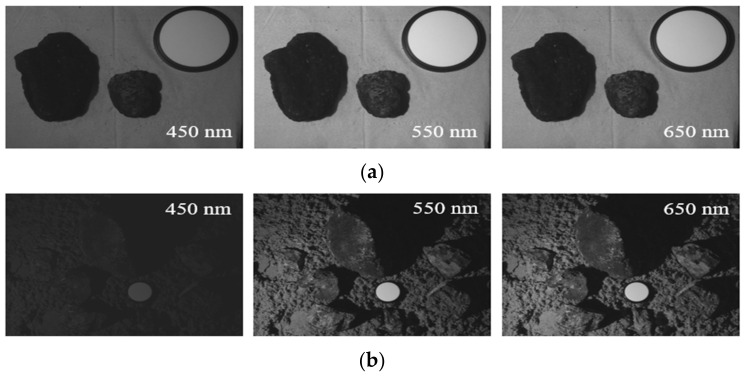

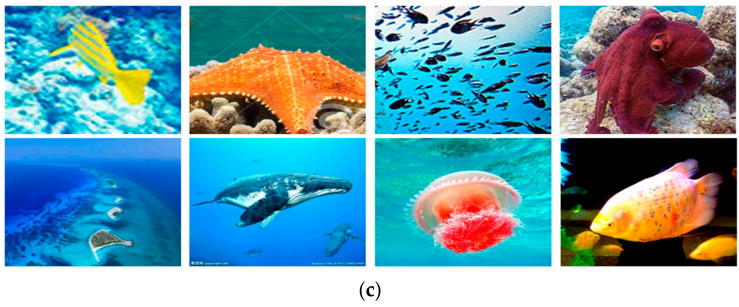
Negative sample images: (**a**) Spectral images of crust; (**b**) Spectral images of minerals and sediments assemblage; (**c**) Negative RGB images that were collected by python crawler.

Semantic segmentation requires pixel-level annotation of images in the dataset. Manual annotation was done using Photoshop CC, with a coral area marked white and background marked as black, as shown in Figure A9. Negative supplementary images, as shown in Figure A10, were used as comparative samples with the images containing corals. Image level annotation, in which images with corals are marked as 1, and images without corals bearing category mark of 0 are also added in the CoralS dataset.

### 2.2. DeeperLabC Model

Unlike other existing semantic segmentation models, the proposed “DeeperLabC” model based on the excellent performance of DeeperLab [43], mainly extracts coral features of a single-channel image and performs semantic segmentation. According to the change in input data dimension, the DeeperLabC model adjusts the number of convolution kernels to meet single-channel image processing requirements. For semantic segmentation, the original model is pruned, and the irrelevant modules such as example segmentation are removed, and the semantic segmentation part is kept, which makes the model more concise. Besides, for the binary classification of coral segmentation, the feature graph’s dimension is reduced, making the size of the model reduced, with a lower amount of memory usage and improved speed of calculations.

**Figure 1:**
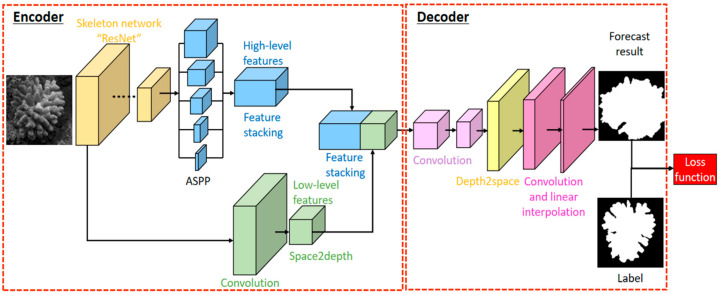
Structure of the DeeperLabC model for coral semantic segmentation.

The structure of DeeperLabC model shown in Figure 1 is divided into encoder and decoder. Initially, the semantic feature maps of the input image to be segmented are obtained by the skeleton network. Low-level features with more texture information are further down-sampled by convolution and Space2Depth (S2D) operations. High-level features are extracted from different receptive fields by Atrous Spatial Pyramid Pooling (ASPP). At the end of the encoder, low-level features after convolution and high-level features extracted by ASPP are stacked on the channel dimension. In the decoder, after a series of convolution operations, the depth to space (D2S) and linear interpolation are used to up-sample the feature map to the size of original image.

**Figure 2:**
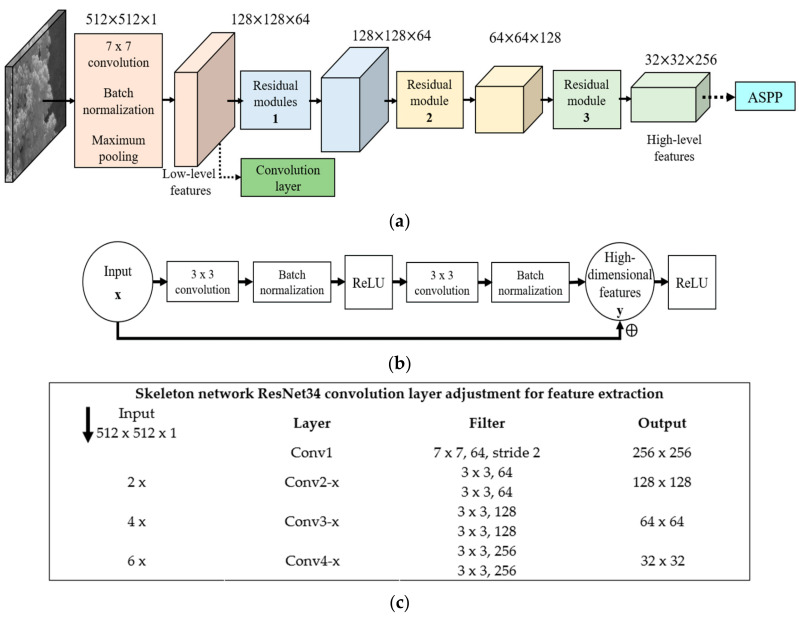
Components of DeeperLabC: (**a**) ResNet34 framework; (**b**) Residual module structure; (**c**) Details of convolution layers of adjusted ResNet34 for feature extraction.

Figure 2 shows the adjusted ResNet34 with residual modules, where the input image is processed by the first layer convolution, batch normalization, and maximum pooling to get the low-level features, which are input into the subsequent convolution layer. To cope with the high processing and large number of deep network parameters, the original ResNet34 model is fine-tuned by pruning the last residual block while maintaining the classification accuracy of the network. The resultant skeleton network contains three residual blocks with 26 convolution layers. After three residual modules, the high-level features are obtained with dimensions reduced to 1/16 of the original input, but the number of channels increased from 1 to 256. Residual block convolutional layers are presented in Figure 2b,c.

Mathematically, the obtained high-dimensional feature y can be expressed as

y = F(x) + x(1)

**Figure 3:**
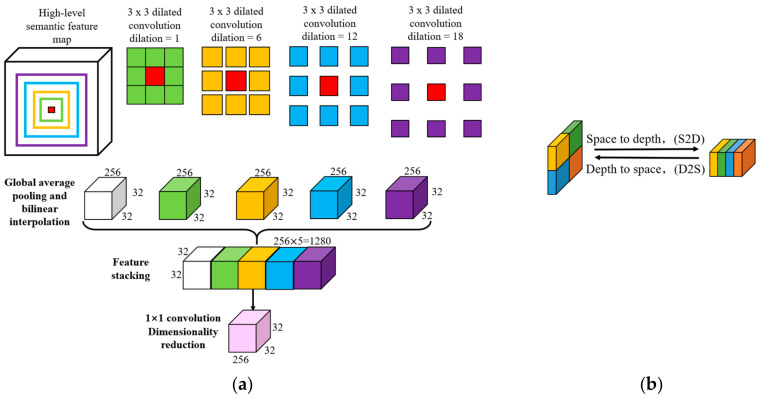
Network modules: (**a**) Atrous Spatial Pyramid Pooling (ASPP) module in DeeperLabC; (**b**) S2D and D2S transformation of the feature map.

The ASPP module takes high-level features from the skeleton network and uses several dilated convolutions with different dilation coefficients for multi-scale sampling in parallel. As shown in Figure 3a, for the same red anchor, the greater the dilation coefficient of the dilated convolution is, the larger the regional characteristics will be calculated and analyzed. In the ASPP module, the global average pooling (GAP) is used to obtain the global features of the input high-level semantic feature map, and the bilinear interpolation is used to enlarge the global features. Four isometric feature maps obtained by four different expansion coefficients through GAP are stacked in channel dimension. Later, 1 × 1 convolution is used to reduce the dimension to 256 to minimize graphics memory consumption and enhance calculation speed.

To handle the GPU memory usage and processing speed of the model while keeping the promising accuracy of the model, Space2Depth for down-sampling and Depth2Space for up-sampling, as in Figure 3b, is used in encoder and decoder, respectively. In the two transformations, each feature map’s volume remains unchanged, only the spatial and channel dimensions are adjusted to achieve up-sampling or down-sampling. To maintain larger receptive fields, DeeperLabC uses a larger kernel size after concatenating low-level and high-level features.

To evaluate the semantic segmentation results, cross-entropy is used as a loss function, and gradient descent is used to optimize the network’s training parameters. Cross entropy function is used to evaluate the probability distribution $y_{p}$ of prediction results and $y_{\mathrm{gt}}$ of real segmentation results, which can be expressed as

(2) $$H\left(y_{\mathrm{gt}}{,\;y}_{p}\right)\;=\;-\sum y_{\mathrm{gt}}\;\cdot \;\log\left(y_{p}\right)$$

## 3. Experimentation

### 3.1. Data Preprocessing

**Figure 4:**
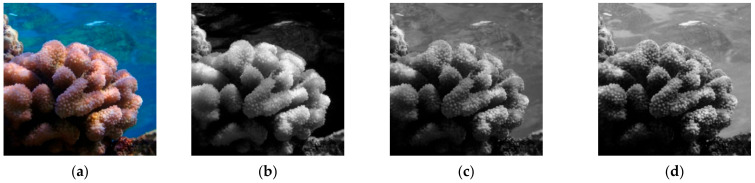

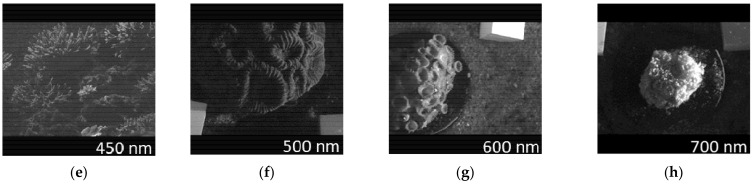
Coral images preprocessing: (**a**) RGB image; (**b**) R channel; (**c**) G channel; (**d**) B channel; (**e**–**h**) Preprocessed spectral images.

Before training the network, all the CoralS dataset images are preprocessed to ensure image size and channel number consistency. Each RGB image is divided into three single-channel images (R, G, B) to keep the channel dimension consistent with the spectral image. To preserve all the features in a spectral image, the black edges at upper and lower ends are added with the aspect ratio of 1:1. All the RGB images, corresponding manually labelled images, and spectral images were uniformly clipped and scaled to the size of 512 × 512 × 1 pixels, as in Figure 4.

### 3.2. Skeleton Network Pre-Training

The skeleton network ResNet34 is pre-trained with an image-level classification label in the CoralS dataset to enhance the model’s feature extraction ability. Quantitative analysis of the dataset for pre-training is presented in Table 3.

**Figure 5:**
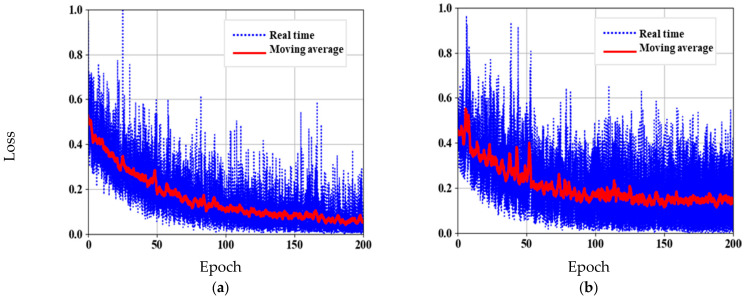
Skeleton network pre-training: (**a**) Training loss curve; (**b**) Validation loss curve.

All images in the training set are augmented randomly with 50% probability; 0–180° random rotation, random horizontal flip, and random vertical flip. While no data augmentation is performed on the images of the validation set. Adam optimizer is used to optimize the parameters, and the initial learning rate is $v_{o}=1\;\times \;10^{-4}$. Exponential decay is used in the learning rate optimization strategy, i.e.,

(3) $$v\;={\;\alpha }^{j}\;\cdot {\;v}_{o}$$

where α is the attenuation coefficient of the learning rate, which is set as constant 0.99, and j represents the number of epochs. The network is trained with total epochs of 200 and a batch size of 32, which took around 10 h to reach the maximum iteration. During training, the network loss is quantified by cross-entropy, and loss curves are plotted as in Figure 5. Training loss and validation loss converges at 0.08 and 0.18, respectively. From the training graph it is revealed that, with the sample dataset, the model reduces the overfitting and increase the generalization in the network.

### 3.3. DeeperLabC Model Training

The data used for training the DeeperLabC model is presented in Table 4, which contains labelled images in CoralS dataset, including single-channel RGB images and spectral images illuminated with white light.

**Figure 6:**
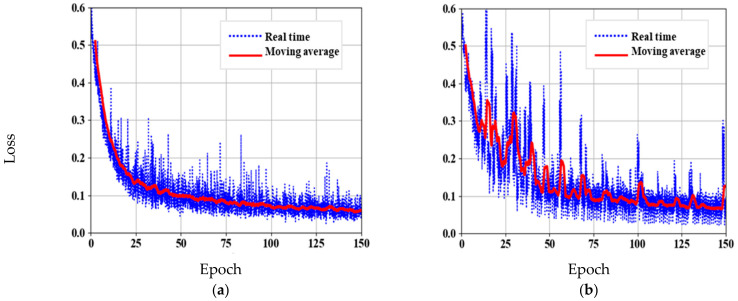
DeeperLabC model training. (**a**) Training loss curve. (**b**) Validation loss curve.

The same data augmentation criteria are performed as that of skeleton network pre-training. The parameters of ResNet34 are loaded, and parameters of DeeperLabC model are adjusted and optimized using Adam optimizer. Keeping the same learning rate, the network took about 5 h to train with 150 epochs and a batch size of 16. The loss curves of DeeperLabC model are shown in Figure 6.

## 4. Results and Discussion

### 4.1. Visualization and Analysis of Segmentation Routine

**Figure 7:**
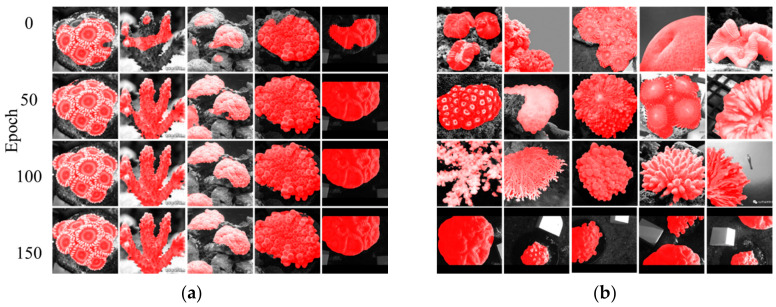
Result visualization: (**a**) Segmentation at different epochs; (**b**) Segmentation of validation set.

The model’s semantic segmentation is visualized at progressing epochs from 0th to 150th, and coral regions are marked with a red mask as in Figure 7a. The network showed improvement in the segmentation of validation set through continuous optimization of network parameters. The parameters of 110 epoch are selected and loaded into the segmentation model, and the validation set images verify the segmentation results. Although the shapes of corals are different, the DeeperLabC model after 110 epochs of training can fairly distinguish between coral and non-coral areas, as in Figure 7b.

### 4.2. Performance Evaluation of Segmentation Model

This section defines the commonly used matrices for evaluation of segmentation model. The evaluation matrices used in this work are given as follows:

(4) $$\mathrm{Precision}\;\left(\mathrm{PR}\right)\;=\;\frac{\mathrm{TP}}{\mathrm{TP}\;+\;\mathrm{FP}}$$

(5) $$\mathrm{Recall}\;\left(\mathrm{RE}\right)\;=\;\frac{\mathrm{TP}}{\mathrm{TP}\;+\;\mathrm{FN}}$$

TP, FP, and FN represent true positive, false positive, and false negative, respectively. F1-score which is the hormonic mean of precision and recall, can be calculated as

(6) $$F1-\mathrm{score}\;=\;\frac{2\mathrm{PR}\;x\;\mathrm{RE}}{\mathrm{PR}\;+\;\mathrm{RE}}$$

Mean IoU is calculated as

(7) $$\mathrm{mean}\;\mathrm{IoU}\;=\;\frac{1}{c\;+\;1}\;\cdot \;\overset{c}{\underset{i=0}{\sum }}\frac{\mathrm{TP}}{\sum_{j=0}^{c}\mathrm{FN}\;+\;\sum_{j=0}^{c}\mathrm{FP}-\mathrm{FN}}$$

**Figure 8:**
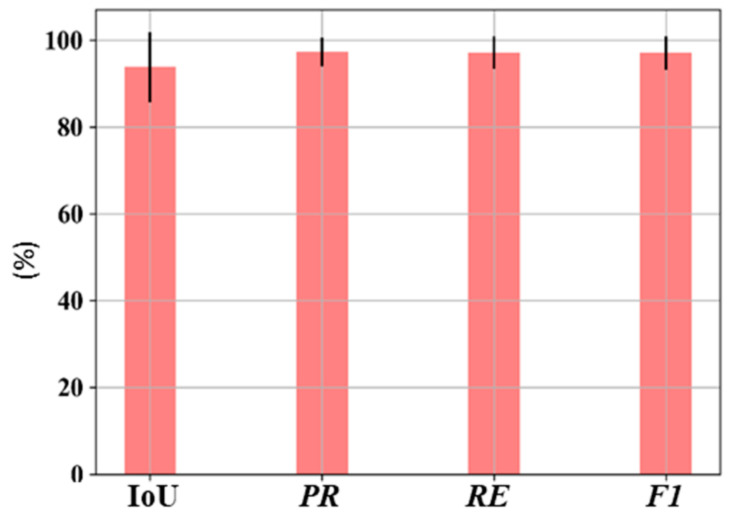
Statistical analysis of DeeperLabC on the validation set.

In the above equation c represents the number of classes. Since this work presents binary classification, the value of c is taken as 2. Statistical analysis of DeeperLabC is presented in Figure 8. The pink column represents each statistical index’s average value, and the black line is the standard deviation. The mean value of IoU was 93.90%, and the average values of PR, RE, and F1-score were 97.31%, 97.13%, and 97.10%, respectively.

### 4.3. Visualization of Segmentation Based on CAM

**Figure 9:**
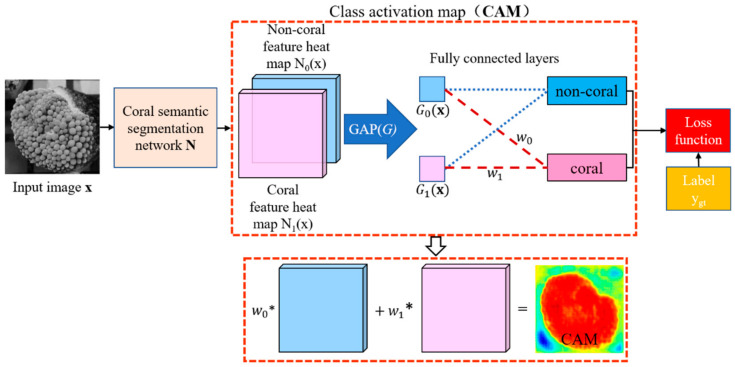
The class activation map (CAM) module in the semantic segmentation network.

CAM module [44] is added to the DeeperLabC model’s tail to visualize the feature map of semantic segmentation. The parameters of network and CAM module are fine-tuned by using the images with category labels in the CoralS dataset. The addition of CAM to the network is described in Figure 9, where $N_{0}\left(x\right)$ and $N_{1}\left(x\right)$ represent a non-coral and coral characteristic thermal map of equal size, respectively. $G_{0}\left(x\right)$ and $G_{1}\left(x\right)$ are the average values of two thermal maps extracted by GAP as

(8) $$G_{j}\left(x\right)\;=\;\frac{1}{h\;\cdot w}\;\cdot \;\sum N_{j}\left(x\right)\;;\;j\;=\;0,\;1$$

where h and w are pixels height and width of the feature images, respectively.

The average values with the weights w0 and w1 predict that the input image belonged to non-coral or coral. Comparing with the real label $y_{\mathrm{gt}}$, the cross-entropy loss function is used to calculate the loss error. The activation map of coral species is calculated as

(9) $$\mathrm{CAM}\left(x\right){\;=\;w}_{0}\;\cdot {\;N}_{0}\left(x\right)\;+{\;w}_{1}\;\cdot {\;N}_{1}\left(x\right)$$

The probability of coral on each pixel is obtained by normalizing CAM, as follows:

(10) $$\mathrm{CAM}\text{’}\left(x\right)\;=\;\frac{\mathrm{CAM}\left(x\right)-\min\left(\mathrm{CAM}\left(x\right)\right)}{\max\left(\mathrm{CAM}\left(x\right)\right)-\;\min\left(\mathrm{CAM}\left(x\right)\right)}\;\times 100\%$$

**Figure 10:**
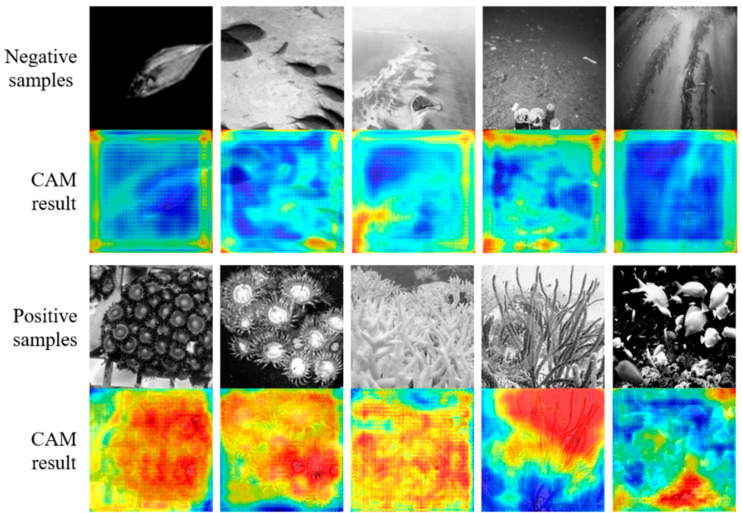
Validation set samples and activation maps.

The same data set as a skeleton network pre-training is used for training, and the parameter matrix of the CAM module is adjusted to get a more accurate activation map. In CAM as in Figure 10, closer to red, shows the higher probability that the pixel belongs to coral, conversely, closer to blue represents a lower probability of belonging to coral. In the negative samples, most of the CAM areas are at a low value; while in the positive samples, the coral area has a more apparent red color, which shows that the proposed DeeperLabC model can better identify the coral features for semantic segmentation.

### 4.4. Comparison with Other Segmentation Models

**Figure 11:**
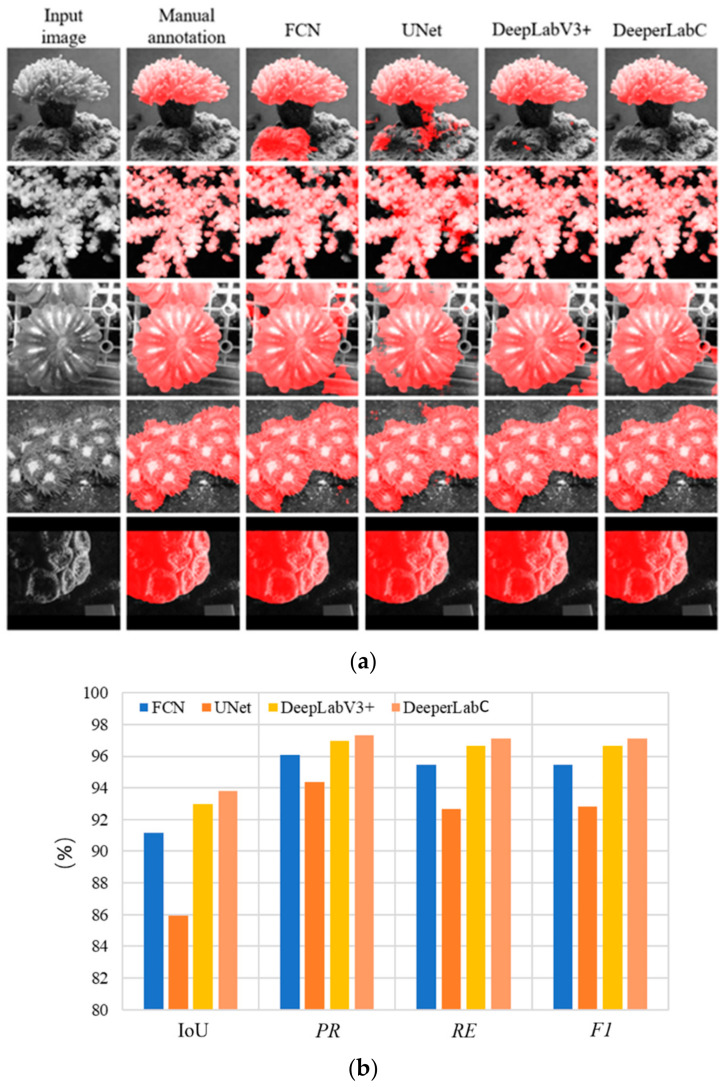
Comparison of semantic segmentation results of different CNN models: (**a**) Segmentation results visualization; (**b**) Statistical comparison of CNN models for semantic segmentation.

FCN, UNet, DeepLabV3+ also have an excellent effect on semantic segmentation; thus, DeeperLabC is compared with these networks, keeping even training criteria for all the networks. Since the dataset includes single-channel images, the dimensions and convolution kernel of the networks are adjusted accordingly. Except for UNet, other networks are loaded with pre-trained ResNet34 as a skeleton network. Using data in Table 3, the networks are trained with exponential decay learning strategy, with learning rate $v_{o}=1\;\times \;10^{-4}$, and Adam as an optimization tool. Segmentation visualization and statistical comparison of the networks are in shown Figure 11.

**Figure A11:**
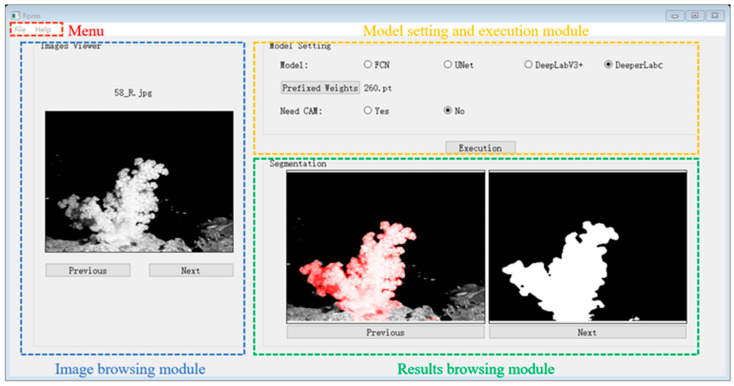
GUI interface for coral semantic segmentation network.

All the models were trained and tested over NVIDIA GeForce GTX 1070ti (8G). Results revealed that all the models provided swift segmentation with a marginal difference in inference time. Proposed DeeperLabC consumed around 1 gigabyte memory of GPU, slightly lower than memory consumed by FCN, UNet, and DeepLabv3+. Lastly, the trained model is encapsulated, and the graphical user interface (GUI) shown in Figure A11, is developed to facilitate the subsequent use. GUI interface is designed and developed based on python 3.5 and Qt 5.14.

## 5. Conclusions

This study presented a CNN-based deep learning model, “DeeperLabC” for semantic segmentation of corals. For the purpose, a distinct coral image dataset which includes single-channel images of various corals was constructed using RGB and spectral imaging technologies. Spectral images at varying wavelengths and RGB images of several coral species under different illumination were collected and used to train and validate the model. The proposed model utilized ResNet34 backbone architecture to extract coral features from single-channel coral images and perform semantic segmentation. In DeeperLabC, convolutional kernels were adjusted for single-channel input images, and the model was fine-tuned, which resulted in a concise semantic segmentation model. The training curves and segmentation visualization results revealed that the model better converged on sample CoralS dataset. The number of training samples in the dataset was sufficient to improve model performance and reduce spurious correlations in dataset that can cause overfitting.

Upon visualizing the segmentation results and analyzing the statistical data after experimentation, the DeeperLabC model proved to be the finest for coral segmentation, compared with FCN, UNet, and DeepLabV3+ model, achieving the resultant mean IoU of 93.90%. Supplementing CAM module to the model provided an additional means of coral segmentation visualization. Finally, the user-friendly GUI module was designed, which encapsulated the entire model for performing semantic segmentation of coral images, visualization of segmentation results, and comparing different neural networks for segmentation.

In literature, remote sensing techniques have stated that corals are dying drastically [45,46]. Our presented system containing coral imaging and the concise and encapsulated system for coral segmentation can be practically utilized for closer and long-term investigation of underwater corals. Future work will focus on enlarging the dataset by adding images of more complex and sparse coral structures. For the detailed morphological study of corals, imaging in other spectral regions such as near-infrared (NIR) will be trialed. Also, the model will be experimented with and deployed for the semantic segmentation of other underwater bodies.

## Figures and Tables

**Table 1:** Dataset collection for coral semantic segmentation.

Location	Coral Species	Reference Figure
Third Institute of Oceanography, MNR, Fujian, Xiamen.	*Plerogyra sinuosa, Acropora* sp.	Figure A1
Institute of Deep-sea Science and Engineering, Chinese Academy of Sciences, Hainan, Sanya.	Dead coral skeleton, *Acropora* sp.	Figure A2
Ocean Optics Laboratory of Zhejiang University, Zhejiang, Zhoushan.	*Trachyphyllia Geofroyi*, *Turbinaria peltate*, *Zoanthus* sp.	Figure A3
Ocean Optics Laboratory of Zhejiang University, Zhejiang, Zhoushan.	Dead coral skeleton, *Montipora Capricornis*, *Trachyphyllia Geofroyi*, *Montipora* *digitate*, *Caulastrea furcate*, *Hydnophora* *exesa*, *Nephthyigorgia* sp.	Figure A4
Shenzhen Da’ao Bay, Coral conservation base about 8 m depth. 22°33′47″ N and 114°27′37″ E	Mainly *Acropora* sp.	Figure A5 and Figure A6

**Table 2:** Quantitative analysis of coral classification dataset.

	Spectral Images	RGB Images	RGB Images from Web Crawler	Total
Positive sample	144	2128	400	2672
Negative sample	150	1209	100	1459
Total	294	3337	500	4131

**Table 3:** ResNet34 pre-training data analysis.

	Training Set 90%	Validation Set 10%	Total
Single channel RGB image	10,359	1152	11,511
Spectral image	174	20	194
Total	10,533	1172	11,705

**Table 4:** DeeperLabC training data analysis.

	Training Set 90%	Validation Set 10%	Total
Single channel RGB image	1552	176	1728
Spectral image	1369	153	1522
Total	2921	329	3250

**Figure A1:**
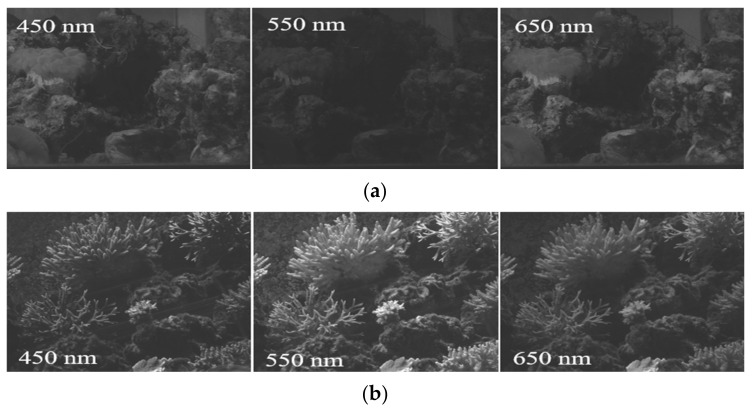
Examples of spectral images under white light. (**a**) *Plerogyra Sinuosa*. (**b**) *Acropora* sp.

**Figure A2:**
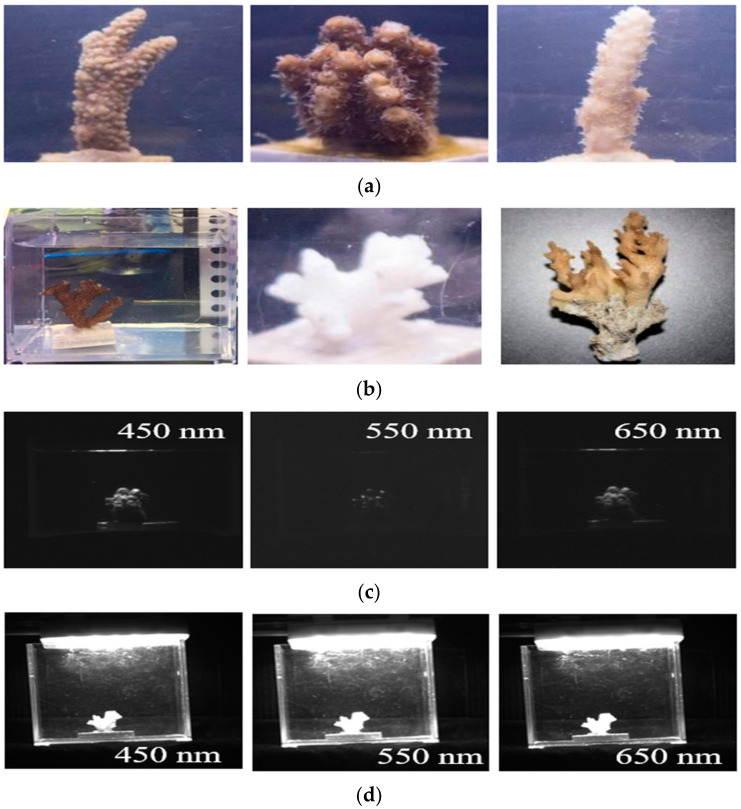
(**a**) RGB images of *Acropora* sp. (**b**) RGB images of dead coral bones. (**c**) Spectral images of *Acropora* sp. illuminated by blue light. (**d**) Spectral images of coral bones under white light.

**Figure A3:**
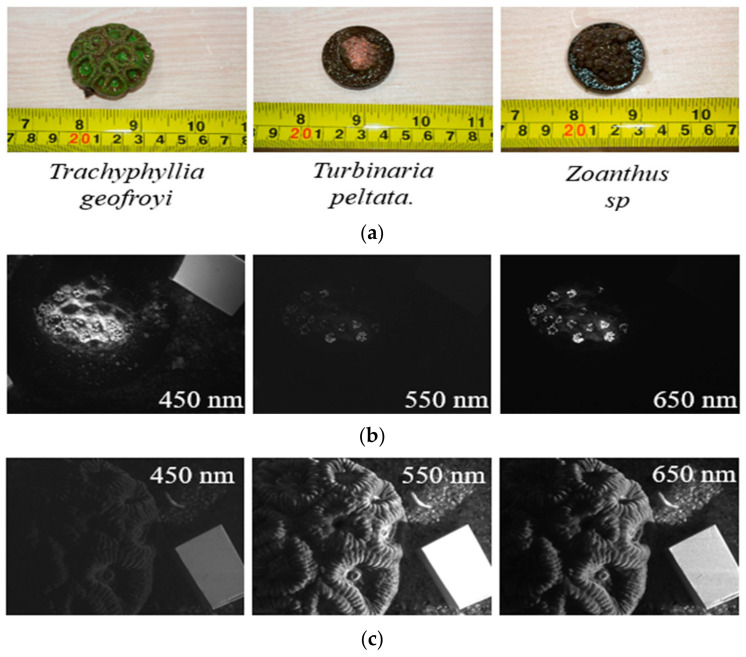
(**a**) RGB images indicating the specie size. (**b**) Spectral images of *Turbinaria Peltata*, illuminated by blue light. (**c**) Spectral images of *Trachyphyllia Geofroyi* illuminated by white light.

**Figure A4:**
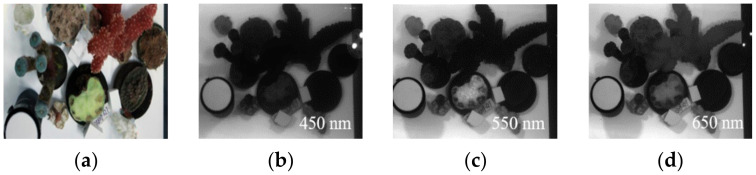
(**a**) Coral species including the dead coral skeleton; (**b**–**d**) Spectral images under white light illumination.

**Figure A5:**
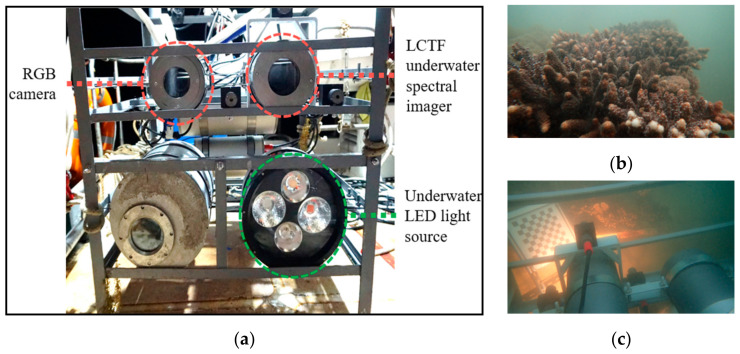
(**a**) Underwater spectral imaging system; (**b**) Imaging *Acropora* sp; (**c**) Imaging site.

**Figure A6:**
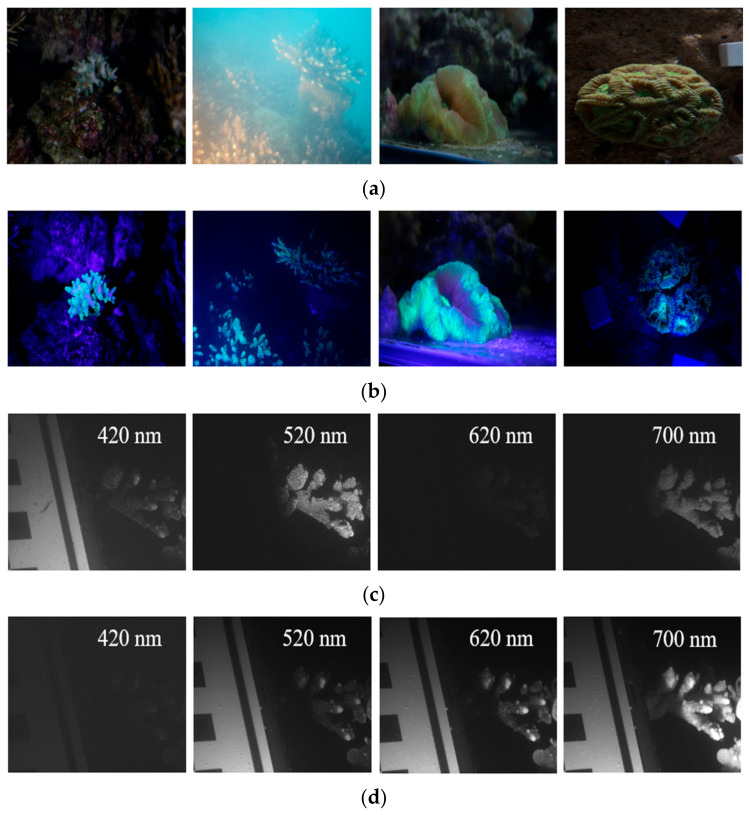
Coral images during the sea trial: (**a**) RGB images under white light; (**b**) RGB images under blue light illumination; (**c**) Spectral images under blue light; (**d**) Spectral images under white light.

## Data Availability

The codes and dataset used in this study are publicly available at https://github.com/YcShentu/CoralSegmentation (accessed on 29 December 2020).
